# Wound Healing Patterns of PRF and PRGF Versus a Collagen‐Based Substitute and Spontaneous Healing—A Randomised Controlled Clinical Trial Assessing Wound Closure of an Experimental, Standardised Palatal Defect

**DOI:** 10.1111/jcpe.70164

**Published:** 2026-08-05

**Authors:** Stefan P. Bienz, Anne C. Fischer, Leonardo Mancini, Franz J. Strauss, Raphael M. Ferrari, Sonja Hitz, Ronald E. Jung, Daniel S. Thoma

**Affiliations:** ^1^ Center for Dental Medicine University of Zurich Zurich Switzerland; ^2^ Discipline of Periodontics School of Dentistry, Faculty of Medicine and Health, The University of Sydney Sydney New South Wales Australia; ^3^ Universidad Autonoma de Chile Santiago Chile; ^4^ Department of Cranio‐Maxillo‐Facial and Oral Surgery University of Zurich Zurich Switzerland

**Keywords:** Autologous platelet concentrates, patient‐reported outcome (PRO), patient‐reported outcome measure (PROM), plasma rich in growth factors (PRGF), platelet‐rich fibrin (PRF), soft‐tissue substitutes, wound healing

## Abstract

**Aims:**

To evaluate wound‐healing progression among platelet‐rich fibrin (PRF), plasma rich in growth factors (PRGF), a collagen‐based wound dressing (CS = collagen substitute) and spontaneous healing (control), assessed by percentage of remaining open wound area and patient‐reported pain.

**Materials and Methods:**

Twenty participants each received four standardised palatal wounds (6 mm × 3 mm) and were randomly assigned to PRF, PRGF, a collagen substitute or spontaneous healing as control. All dressings were secured with criss‐cross sutures. Standardised clinical photographs and pain assessments were obtained at baseline and on postoperative days 2,5,7,9, 12 and 14. Parametric and nonparametric models for longitudinal data were used to compare outcomes, accounting for within‐subject correlation of repeated measurements.

**Results:**

Open wound area decreased across all groups throughout the 14‐day period. Some defects enlarged transiently at Day 2, and the greatest differences between the groups were observed on Day 7 (35.7%–47.0% of wound remaining). Pain scores decreased significantly over time in all groups, with no statistically significant differences between treatments (*p* > 0.05). Anterior defects tended to be perceived as more painful than posterior defects.

**Conclusions:**

Spontaneous healing resulted in slightly faster reduction of remaining open wound area than PRF, PRGF or the collagen substitute, with comparable postoperative pain levels. In standardised defects in healthy individuals, PRF, PRGF and collagen substitutes demonstrate minimal additional benefit for soft‐tissue healing. Further studies are warranted to determine whether benefits emerge in larger wounds or in patients with compromised healing.

## 1 Introduction

Mucogingival surgeries have become routine procedures to enhance the quality and/or quantity of soft tissues around teeth and implants. Systematic reviews have revealed the benefits of these procedures in establishing favourable conditions to maintain peri‐implant health and to improve aesthetics (Thoma and Strauss 2022; Zhang et al. 2025). However, the interventions are associated with postoperative morbidity (Hämmerle et al. 2023; Thoma et al. 2023), and there is a risk for complications, which can negatively affect treatment outcomes (Stefanini et al. 2021).

To limit these complications during wound healing, various adjunctive strategies have been proposed, including the application of wound dressings on open oral wounds. Collagen‐based wound dressings have been shown to improve early wound healing compared to spontaneous healing (Thoma et al. 2012). Among the available options currently used, platelet‐rich fibrin (PRF) and plasma rich in growth factors (PRGF) have gained increasing attention by clinicians and the scientific community (Avila‐Ortiz et al. 2022; Miron et al. 2025; Siawasch et al. 2025; Strauss et al. 2018).

In vitro, PRF/PRGF promotes cell proliferation, migration and differentiation (Strauss et al. 2020) and exerts anti‐inflammatory effects (Nasirzade et al. 2020). Clinically, systematic reviews have documented their use in implant dentistry (Strauss et al. 2018), where they have been proposed to support soft‐tissue healing (Miron et al. 2016). However, the clinical evidence remains inconsistent. An earlier randomised controlled trial (RCT) (Temmerman et al. 2016) suggested that PRF may be beneficial for alveolar ridge preservation. In contrast, more recent split‐mouth RCTs failed to confirm these advantages (Abad et al. 2023; Wang et al. 2022).

Wang et al. reported no improvements in soft‐tissue healing or alveolar ridge dimensions when PRF was used compared to spontaneous healing (Wang et al. 2022). These findings have been corroborated by additional recent RCTs showing no clear benefit of PRF for ridge preservation (Abad et al. 2023).

Beyond potential regenerative effects, PRF and PRGF have also been proposed to reduce postoperative pain (Temmerman et al. 2016). One RCT showed that PRF/PRGF can reduce postoperative pain after tooth extraction (Temmerman et al. 2016). Similarly, a more recent systematic review concluded that PRF may reduce postoperative pain, enhance soft‐tissue healing and limit dimensional bone loss, particularly during the early healing phase of 2–3 months (Al‐Maawi et al. 2021). This potential effect is clinically relevant, given the paradigm shift in regenerative dentistry towards patient‐reported outcomes (PROs) (McGrath 2014; McGrath et al. 2012; Schmitt et al. 2019; Thoma and Strauss 2022). The main value of PROs lies in considering patients' preferences and perceptions. This may help determine whether a given intervention meaningfully improves their postoperative experience (Chalmers and Glasziou 2009). In fact, reliance on patients' preferences is becoming increasingly important for the selection of therapy (McGuire et al. 2014).

Wound closure is a commonly used and clinically meaningful parameter for evaluating healing progression. It enables quantification of tissue regeneration and early detection of complications (Gould and Li 2019). Wound healing is a rapid and complex biological process, as a short time frame coincides with key biological events such as inflammation resolution, granulation tissue formation and early epithelialisation. The evaluation of several time points and the use of more advanced statistical models may help to better understand wound healing (Cukjati et al. 2001).

Autologous platelet derivates have been widely investigated in soft‐tissue healing because of their sustained release of growth factors, fibrin scaffold formation and immunomodulatory properties (Kang et al. 2011; Strauss et al. 2020; Zwittnig et al. 2022). Although several studies report enhanced angiogenesis and epithelialisation, clinical outcomes remain heterogeneous (Canellas et al. 2020; Miron et al. 2016). In contrast, collagen‐based wound dressings primarily function as structural scaffolds and protective barriers (Sai K & Babu, 2000). They stabilise the clot, support cell migration and maintain a moist wound environment but do not provide autologous growth factors or active biological signalling (Brett 2008). As collagen substitutes are widely used in clinical practice, they represent a clinically relevant comparator with predominantly passive biological activity (Alberts et al. 2025).

Therefore, the aim of the present study was to compare wound‐healing progression among PRF, PRGF, a collagen‐based wound dressing (CS, collagen substitute) and spontaneous healing (control). Outcomes were assessed by (i) wound closure, expressed as the percentage of the remaining open wound area, and (ii) patient‐reported pain. The primary endpoint was the percentage of the remaining open wound area, where 100% representing the fully open defect immediately after creation and 0% indicating complete epithelial closure.

## 2 Material and Methods

### 2.1 Study Design and Population

This randomised controlled trial employed a within‐subject experimental design and received approval from the competent Ethics Committee (Kantonale Ethikkommission, BASEC‐Nr: 2024–00680). It was registered in the German Clinical Trials Register (DRKS00034761). The study adhered to the principles of the Declaration of Shephard (1976, last revised in 2013) and conformed to the CONSORT guidelines. The percentage of remaining open wound was the primary outcome.

Sample size calculation yielded that 20 participants were required. The study cohort primarily comprised individuals motivated to contribute to scientific research and the progress of medical practices.

To prevent repetitive allocation sequences across participants, a permutation‐based randomisation approach was used instead of a simple randomisation. Further details are reported in the Supporting Information: Appendix A.

All surgical procedures and follow‐up evaluations were performed by a single experienced surgeon at the Clinic of Reconstructive Dentistry. Owing to the study design, blinding of the operator and participants was not possible. However, the outcome assessor conducting the photographic analyses was blinded to the three surgical sites treated with wound dressings. The control site, which did not receive any dressing, was easily distinguishable to the assessor.

### 2.2 Eligibility Criteria

Participants were considered eligible for inclusion in the study if all of the following criteria were met: they were male or female individuals between 18 and 80 years of age; were systemically healthy; demonstrated good oral hygiene, indicated by a plaque index of less than 30%; showed no signs of periodontal disease, defined as probing depths not exceeding 3 mm (with the exception of third molars); were fully dentate, except for third molars, with no untreated carious lesions; and possessed adequate space between the first molar and the canine to accommodate two 6‐mm biopsies with a minimum distance of 3 mm between them. Additionally, participants were required to provide written informed consent. Detailed exclusion criteria can be found in the Supporting Information: Appendix A.

### 2.3 Surgical Procedure

Four standardised palatal defects, each with a diameter of 6 mm, were created using a biopsy punch (Stiefel biopsy punch, Stiefel Laboratorium, Offenbach, Germany) following the administration of local anaesthesia (Ubistesin forte 4% 1:100,000, Ubistesin, 3M, Saint Paul, Minnesota, USA). The depth of each defect was measured using a periodontal probe, and the tissue was then excised using a surgical blade (SM67 Swann‐Morton, Sheffield, England) to a depth of 3 mm below the surface. The two distal defects were positioned mesial to the first molars on each side, while the two mesial defects were located in the region of the first premolars. A minimum distance of 3 mm was maintained between each defect and the adjacent teeth, as well as between neighbouring defects, to ensure surgical safety and tissue viability.

**FIGURE 1:**
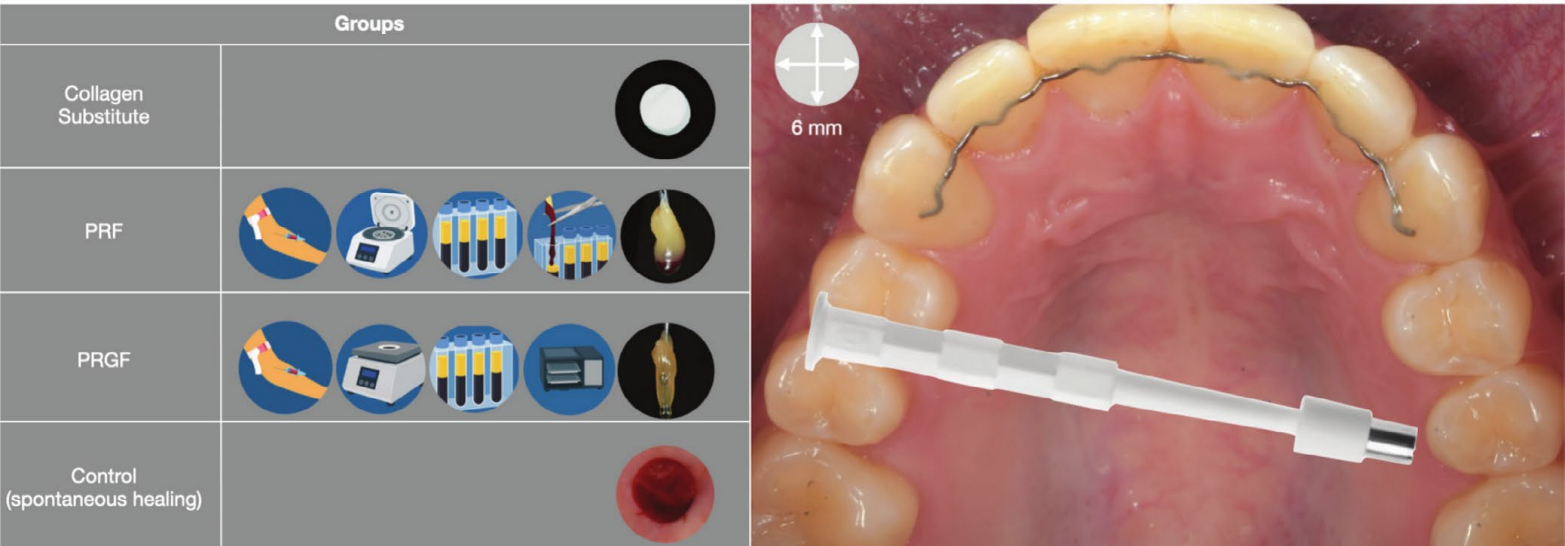
Overview of the study procedure. (Left): Four groups and corresponding preparation of the palatal wound dressings. (Right): Creation of the standardised palatal defects.

**FIGURE 2:**
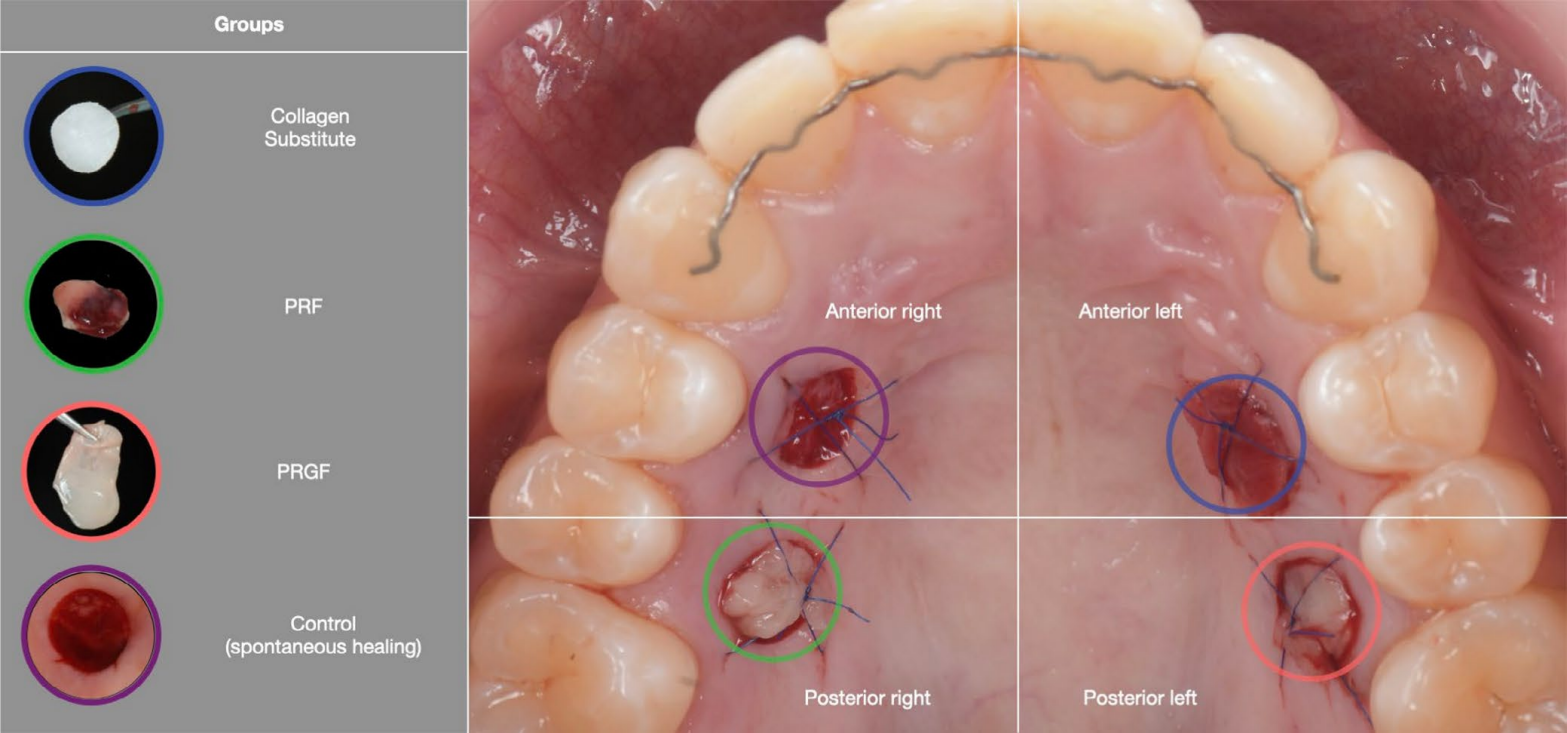
Random group allocation and placement of the palatal wound dressings.

Each defect was randomly assigned to one of the following four treatment modalities (Figures 1 and 2):

1. PRGF (Plasma Rich in Growth Factors)
  PRGF is a regenerative material that utilises the patient's own blood–derived growth factors to promote tissue healing (Anitua et al. 2014). The plasma was prepared through plasmapheresis from venous blood collected into plastic Eppendorf tubes pre‐loaded with 0.45 mL of sodium citrate as an anticoagulant (PRGF‐Endoret tubes, BTI Biotechnology Institute). This clot was then precut, condensed and sutured onto the palatal defect using a criss‐cross technique.
2. PRF (Platelet‐Rich Fibrin)
  Like PRGF, PRF was obtained from the patient's own blood; however, no anticoagulants were used during collection (Temmerman et al. 2016; Wang et al. 2022). This membrane was then precut, placed onto the palatal wound and secured using a criss‐cross suture.
3. CS (Collagen Substitute)
  CS (Geistlich Mucograft, Geistlich Pharma AG, Wolhusen, Switzerland) had a standard diameter of 8 mm and a thickness of 3 mm. A biopsy punch (⌀ = 6 mm) was used to trim the material to the appropriate diameter before placement. Once shaped, the membrane was positioned over the defect and fixed in place with a criss‐cross suture.
4. Control (Spontaneous Healing)
  In this group, the defect was left to heal without a dressing. A criss‐cross suture was applied to stabilise the blood clot and protect the wound.

Details on PRF and PRGF procedures are found in the Supporting Information: Appendix A.

### 2.4 Postoperative Care

Participants were instructed to rinse with a 0.2% chlorhexidine mouthwash (Curasept 0.2%) twice daily, starting 24 h after surgery and continuing until Day 5, when sutures were removed. Ibuprofen Sandoz 600 mg was provided as pain medication, to be taken as needed.

### 2.5 Evaluation of Remaining Open Wound Area (Primary Outcome)

**FIGURE 3:**
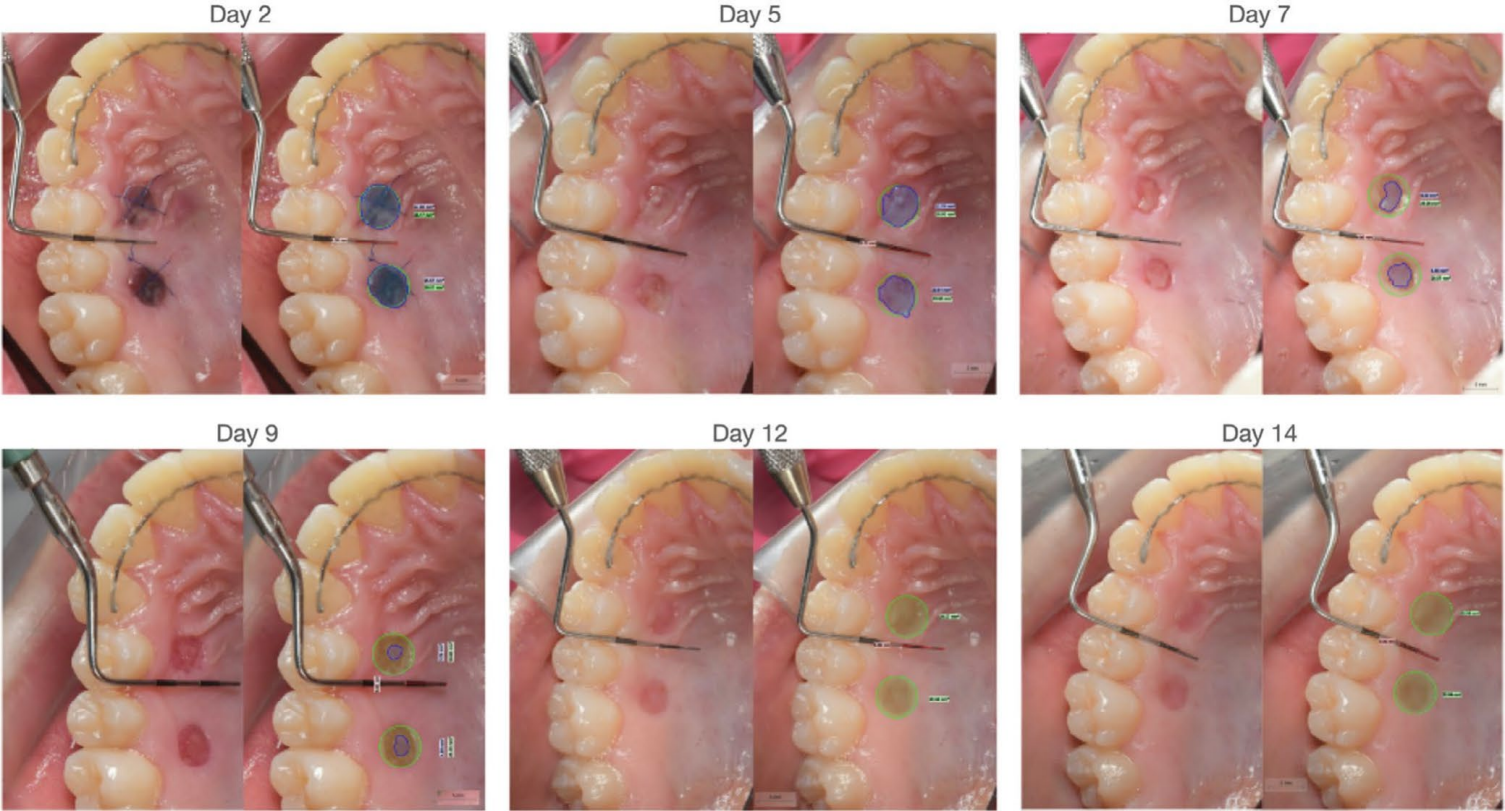
Representative case showing the standardised defects with the adjacent probe for calibration of the measurements over the observation period from Day 2, 5, 7, 9, 12 and 14. The left image shows the defect alone, and the right image shows the superimposed surface measurement of the open wound.

**FIGURE 4:**
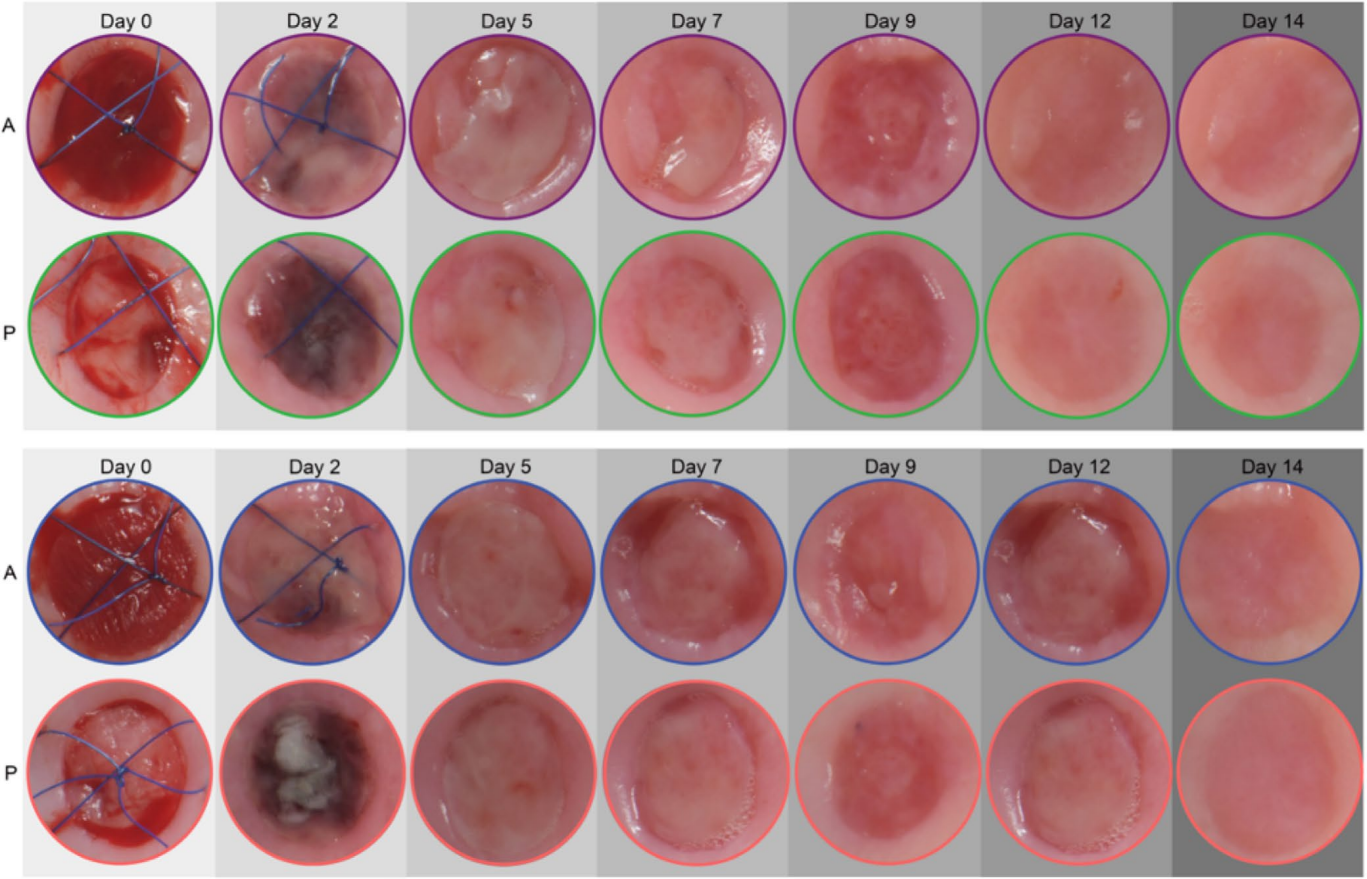
Representative cases from each treatment group showing the standardised palatal defects immediately after application of the assigned treatment and the subsequent healing progression over time. A = anterior; P = posterior; Row 1 = spontaneous healing (control); Row 2 = PRF; Row 3 = collagen substitute; Row 4 = PRGF.

The remaining open wound area was clinically assessed through measurements taken from standardised photographs captured at baseline (prior to treatment) and at 2, 5, 7, 9, 12 and 14‐days post surgery. To calibrate the initial 6‐mm defect size, a periodontal probe was positioned adjacent to each defect, as illustrated in Figure 3. The percentage of remaining open wound area was determined by outlining the borders of the remaining wound and calculating the surface area relative to the original defect size, using an image analysis software (Leica Microsystems, LAS 4.6, Wetzlar, Germany). This approach followed a method comparable to that used in histomorphometry (Thoma et al. 2018). All photographs were taken by the operating surgeon, and the analysis was performed by a single trained histologist. Figure 4 presents the wound healing progression of a representative case for each treatment group.

### 2.6 Patient‐Reported Outcome Measures (PROMs) (Secondary Outcome)

Participants' perception of pain was evaluated using a numerical rating scale (NRS) both prior to the surgical procedure and at each follow‐up appointment. They were asked to indicate their pain level by selecting a value on the NRS, an 11‐point ordinal scale ranging from 0 (no pain) to 10 (worst imaginable pain). Pain levels were assessed separately for each treated site.

### 2.7 Statistical Analysis

Descriptive statistics were calculated, which included means, standard deviations (SD) and medians for metric parameters, as well as frequencies for categorical parameters. Details of analysis and sample size calculation are displayed in the Supporting Information: Appendix A.

## 3 Results

### 3.1 Study Population

A total of 20 participants, aged 20 to 61 years (mean age: 28.5 years, median: 27.0 years, *σ*: 8.4), were screened and enrolled and adhered to the study protocol. Among them, 55% (11 individuals) were female, while 45% (9 individuals) were male. Each participant received four standardised palatal wounds to assess healing dynamics; two sites were treated with autologous blood concentrates (PRF and PRGF), one with a collagen substitute (CS) and one site was left untreated to heal spontaneously (control). One patient was a smoker (< 10 cigarettes/day) and three patients were former smokers. One participant missed two of the follow‐up visits, so wound healing could not be evaluated due to missing photographs. Four patients experienced premature suture loss (Visit 3, Day 2) without loss of the dressing or blood or fibrin clot. No further adverse events were reported.

### 3.2 Clinical Evaluation of Remaining Open Wound (%)

**FIGURE 5:**
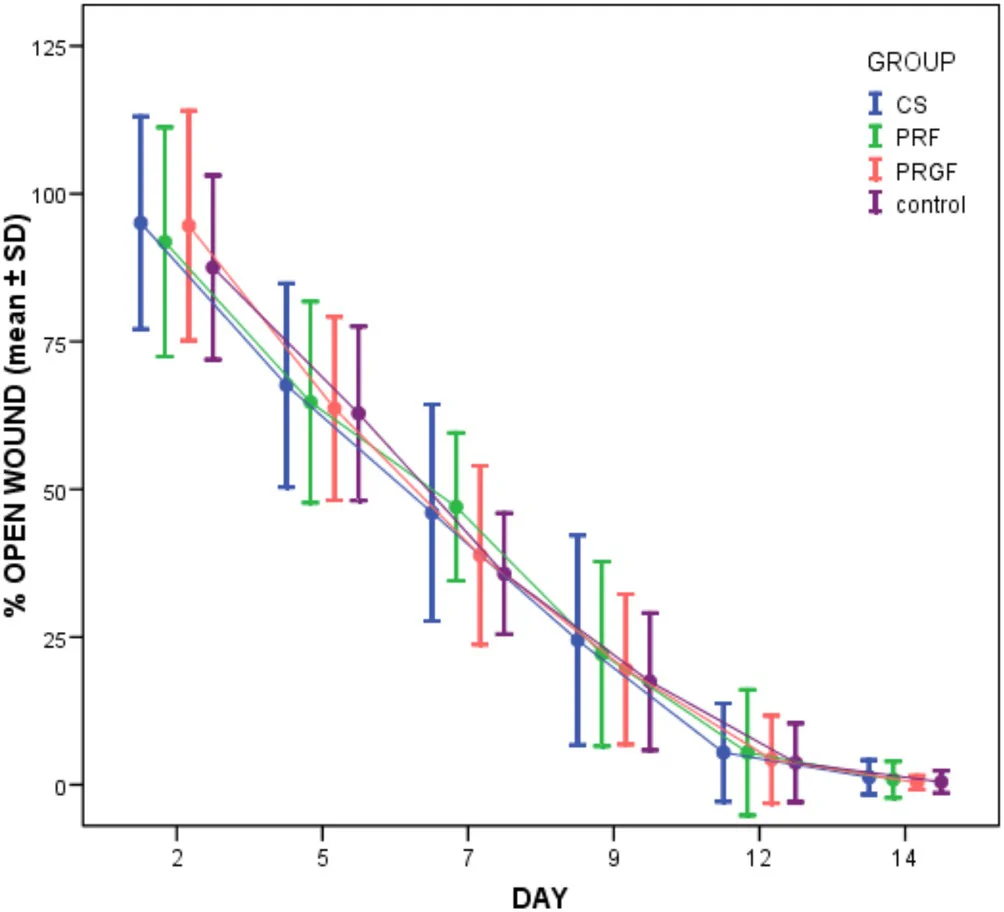
Mean percentage of remaining open wound area across the 14‐day observation period for each treatment group. CS, collagen substitute; PRF, platelet‐rich fibrin; PRGF, plasma rich in growth factor. Control was spontaneous healing. Error bars indicate SD.

The percentage of open wound over the 14‐day observation period is shown in Table 1 and Figure 5. On Day 2, in some individuals of each group, the percentage of open wound exceeded 100%, indicating transient enlargement (the defect was larger than the surgically created wound). The largest inter‐group differences were observed at Day 7. The mean (±SD) open wound percentage was 47.0% (±12.5) for PRF, 38.8% (±15.1) for PRGF, 46.0% (±18.3) for CS and 35.7% (±10.0) for control (spontaneous healing). By Day 14, residual open wound area was minimal in all groups: 0.9% (±3.1) for PRF, 0.4% (±1.2) for PRGF, 1.3% (±2.9) for CS and 0.5% (±1.9) for control, with 85% of wounds achieving complete closure.

**TABLE 1:** Remaining open wound (%) over the entire observation period.

	CS	PRF	PRGF	Control
	Mean ± SD (95% CI)	Mean ± SD (95% CI)	Mean ± SD (95% CI)	Mean ± SD (95% CI)
Day 2	95.1 ± 18.0 (86.6; 103.5)	91.9 ± 19.4 (82.8; 100.9)	94.6 ± 19.4 (85.5; 103.7)	87.5 ± 15.6 (80.2; 94.8)
Day 5	67.6 ± 17.2 (59.5; 75.7)	64.7 ± 17.0 (56.8; 72.7)	63.7 ± 15.5 (56.4 70.9)	62.8 ± 14.7 (55.9; 69.7)
Day 7	46.0 ± 18.3 (37.4; 54.6)	47.0 ± 12.5 (41.2; 52.9)	38.8 ± 15.1 (31.8; 45.9)	35.7 ± 10.2 (30.9; 40.5)
Day 9	24.5 ± 17.7 (16.1 32.8)	22.2 ± 15.6 (14.9; 29.5)	19.5 ± 12.7 (13.6; 25.5)	17.5 ± 11.6 (12.0; 22.9)
Day 12	5.5 ± 8.3 (1.6; 9.4)	5.5 ± 10.6 (0.5; 10.4)	4.3 ± 7.4 (0.8; 7.8)	3.7 ± 6.7 (0.6; 6.9)
Day 14	1.3 ± 2.9 (−0.1; 2.6)	0.9 ± 3.1 (−0.5; 2.3)	0.4 ± 1.2 (−0.2; 0.9)	0.5 ± 1.9 (−0.4; 1.4)

*Note:* Control was spontaneous healing.

Abbreviations: CI, confidence interval; CS, collagen substitute; PRF, platelet rich fibrin; PRGF, plasma rich in growth factor; SD, standard deviation.

**FIGURE 6:**
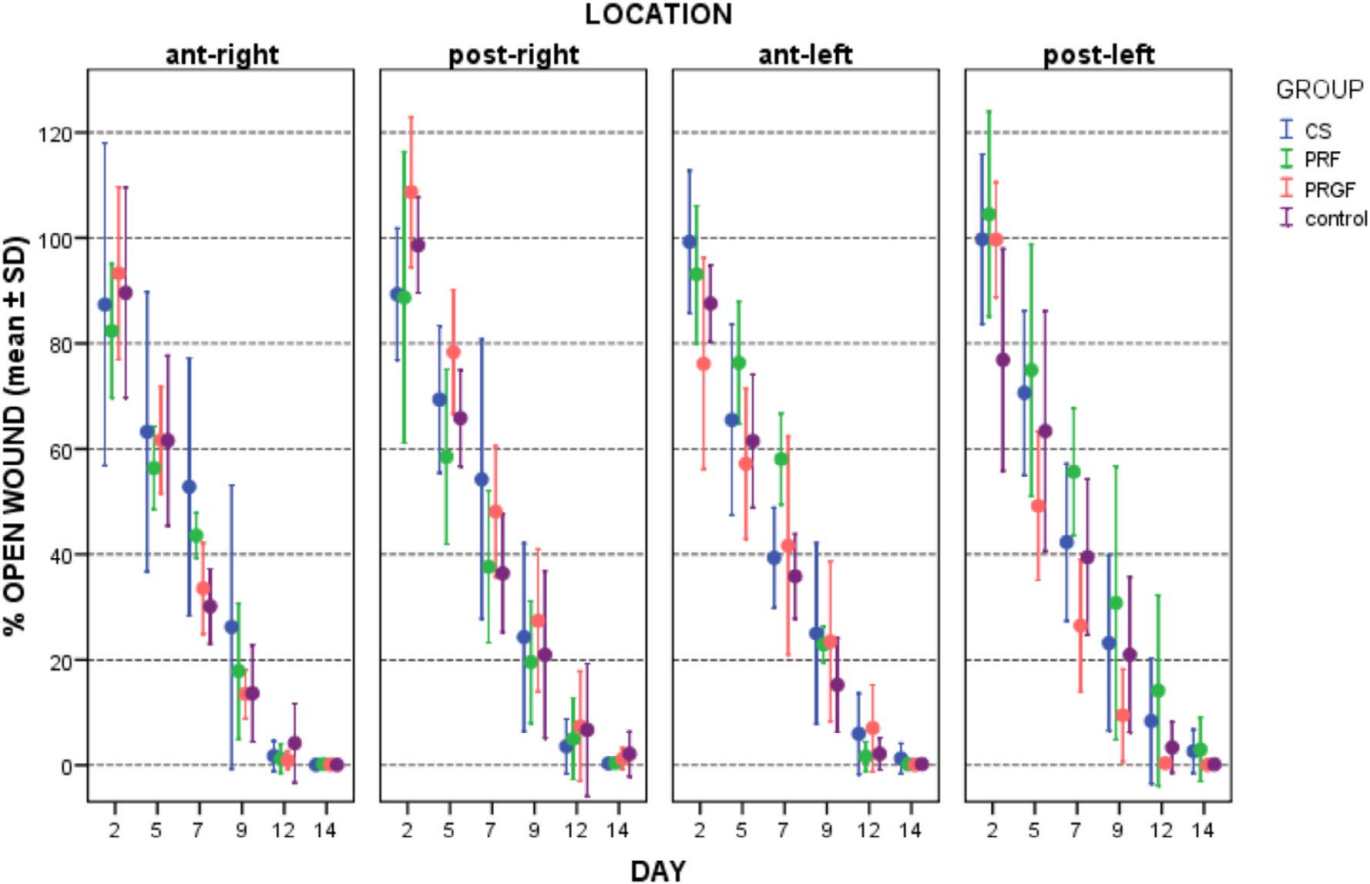
Remaining open wound area by day, treatment group and wound location. Mean percentage of open wound area across visits for each treatment group: CS, collagen substitute; PRF, platelet‐rich fibrin; PRGF, plasma rich in growth factor. Control was spontaneous healing. Error bars indicate SD.

The generalised linear model confirmed that healing patterns were influenced simultaneously by time, treatment group and wound location. There were strong interactions among visit, group and location (visit × group × location, *p* < 0.001), indicating that the effect of any one factor depended on the others (Figure 6). Although the wound area was found to be decreased across all visits, the rate of healing differed between treatment groups and varied by whether the wound was located anteriorly or posteriorly. These findings emphasise that wound healing dynamics cannot be interpreted by treatment group alone but must be understood in the context of both time and anatomical location.

Estimated marginal means showed that, when averaging across all visits and locations, the PRF and CS groups had significantly larger open wound areas compared with control (*p* = 0.018 and *p* = 0.042 respectively, Table S1). The magnitude of these differences was moderate, with effect sizes of *d* = 0.67 for PRF and *d* = 0.60 for CS.

### 3.3 Pain Perception

Pain scores decreased significantly over time (*p* < 0.001) (Figure S1) following a similar trajectory across all four groups (*p* = 0.422). No significant differences in pain were found between treatment groups at any timepoint (*p* = 0.737). However, marginally lower pain was observed for PRGF compared with control at Visit 7 (*p* = 0.084, effect size *d* = 1.32). Wound location tended to influence pain perception (*p* = 0.084, relative effect difference = 0.28); anterior sites were reported as more painful than posterior sites (Figure S2—relative effect).

### 3.4 Pain Killers

Analgesic intake was highest on the day of surgery, with a mean of 1.80 ± 1.51 doses. Usage declined sharply to 0.75 ± 1.48 on postoperative Day 1 and continued to decrease gradually thereafter. By Day 13, the mean intake was 0.05 ± 0.22 doses.

Median consumption was zero from Day 1 onwards, indicating that at least half of the participants took no painkillers after the first postoperative day. By Day 13, a few participants took a single analgesic dose.

## 4 Discussion

The present randomised experimental clinical study predominantly revealed: (1) spontaneous healing resulted in a significantly faster reduction of remaining open wound area compared with PRF and collagen substitute (CS), with no significant differences between PRF and PRGF; (2) pain perception did not differ significantly between treatment groups; (3) anterior sites tended to heal faster and tended to be perceived as more painful than posterior sites; (4) transient swelling was observed at Day 2 across all groups; and (5) no adverse events were noticed in any participant.

The finding that spontaneous healing was faster than healing with PRF, PRGF or CS contrasts with the commonly cited regenerative advantages of autologous platelet concentrates (Calciolari et al. 2025; Miron et al. 2025; Quirynen et al. 2025) and collagen‐based biomaterials. While previous in vitro and preclinical studies have demonstrated that PRF can enhance angiogenesis, fibroblast proliferation and growth factor release (Dohan Ehrenfest et al. 2009; Miron et al. 2017; 2020), the present results align more closely with recent randomised controlled trials reporting no clear improvement in early soft‐tissue healing or alveolar ridge preservation with PRF (Abad et al. 2023; Wang et al. 2022). Several factors may explain this discrepancy. Differences in preparation protocols as well as environmental conditions such as maintaining centrifugation and handling temperatures between 21°C and 30°C (Wu et al. 2023) may introduce variability in the biological quality of platelet‐derived matrices. Additionally, it can be speculated that any material placed on an open defect, whether autologous or collagen‐based, may act as a temporary physical barrier to epithelial migration. In practical terms, epithelial cells may first need to circumvent or remodel the collagen substitute before advancing across the wound surface, thereby delaying re‐epithelialisation relative to spontaneous healing. In contrast, PRF and PRGF function as sustained reservoirs of growth factors. They also provide a fibrin scaffold that reinforces the physiological clot and serves as a provisional matrix, retaining platelets and leukocytes within the local microenvironment (Kang et al. 2011; Strauss et al. 2020). For the translation into clinical indications, the authors suggest that a difference of 15%–20% in this particular experiment at Day 7 (highest difference is usually around Day 7) would be clinically meaningful.

Furthermore, the experimental defects in this study were created in a region with high vascularisation, and therefore a high delivery of growth factors by the surrounding is suspected. It should be noted that, due to the proximity of the placed defects, diffusion of growth factors from PRF and PRGF sites to adjacent control and CS sites cannot be excluded. Therefore, potential biological cross‐interference should be considered and acknowledged as a limitation of the experimental study design. Although platelet concentrates release endogenous growth factors over a period of hours to days, few studies have directly quantified the radial distribution of these factors within surrounding tissues (Richard J Miron et al. 2016). Most studies investigating growth factor release primarily report temporal elution profiles rather than spatial diffusion distances within surrounding tissues (Kobayashi et al. 2016; Zwittnig et al. 2022). The effective signalling range of growth factors in vivo is further limited by rapid binding to extracellular matrix components, receptor‐mediated uptake and proteolytic degradation, indicating that biological activity is typically confined to short distances (generally tens to a few hundred micrometres) rather than extending across several millimetres (Kihara et al. 2013).

Pain perception followed a similar trajectory, and no statistically significant differences were detected. This aligns with prior reports showing inconsistent or limited analgesic benefits of PRF in oral surgical procedures (Del Fabbro et al. 2014). In the present study, anterior sites were associated with slightly higher pain scores (*p* = 0.08), likely reflecting regional anatomical differences. The anterior palate contains a denser distribution of sensory nerve fibres and is more actively engaged during speech and swallowing, which may exacerbate discomfort. Interestingly, despite higher pain scores, anterior wounds showed faster epithelialisation, consistent with known histological features of thinner palatal mucosa and enhanced vascularity in these regions (Squier and Hill 1985).

Swelling observed at Day 2 across all groups is consistent with the expected inflammatory response after soft‐tissue manipulation. Variability in injection technique and the vasoconstrictive effects of local anaesthetics containing adrenaline may also influence early oedema formation. As expected, swelling gradually subsided over the following days, reflecting a normal self‐limiting inflammatory process. Importantly, no adverse effects occurred with PRF or CS, reinforcing their biocompatibility (Dohan Ehrenfest et al. 2012) and predictable resorption behaviour (Solderer et al. 2023).

Collectively, these findings suggest that, under conditions of standardised, clean palatal defects and partial primary closure, natural healing may be highly efficient and may not require additional biomaterials to enhance early epithelialisation or reduce postoperative discomfort. Whether particular patient populations—such as smokers, individuals with diabetes or those with impaired wound healing—might benefit more substantially from PRF, PRGF or collagen substitutes remains uncertain and warrants further research. Likewise, although clinical outcomes were comparable, microscopic or molecular differences in healing dynamics were not assessed and could reveal subtle biological effects not evident clinically.

This study has limitations. Analgesic intake was not standardised, and variations in painkiller use could influence patient‐reported pain scores, potentially masking true differences between groups. Future studies should control for analgesic consumption or include it as a covariate. The standardised 6‐mm palatal defect created in systemically healthy individuals represents a low‐morbidity model with high intrinsic regenerative capacity. In such conditions, rapid physiological healing may mask subtle treatment‐related effects. Additionally, wound healing assessments relied partly on photographic analyses. Although this technique is widely accepted in dermatology and was conducted according to standardised protocols, its limited use in dental research may introduce minor variability. Although the photographic analysis was performed by a single blinded assessor, the control sites were inherently identifiable, resulting in partial unblinding. Given that photographic assessment of remaining open wound area constituted the primary outcome measure, this potential bias should be acknowledged. Finally, the sample size relied on a standardised effect size (Cohen's *d* = 0.7), reflecting a large effect, to detect an absolute difference in open wound area that was visible and clinically perceptible. However, clinically important difference involves a degree of subjectivity which some clinicians may reasonably disagree with. Furthermore, complete blinding was not feasible in the present study because the control site was inherently identifiable. In accordance with CONSORT recommendations, this limitation is acknowledged as a potential source of bias that may have influenced the study results.

## 5 Conclusion

Spontaneous healing resulted in slightly faster reduction of remaining open wound area than PRF, PRGF or collagen substitutes, with comparable postoperative pain levels. In this low‐morbidity model of standardised 6‐mm palatal defects in healthy individuals, adjunctive use of PRF, PRGF or collagen substitutes provided no clinically relevant additional benefit over spontaneous healing. The high intrinsic regenerative capacity of the defects and the predominantly young and healthy study population, together with the primarily localised biological activity of growth factors, may have reduced the ability to detect treatment‐related differences. Accordingly, these findings should be interpreted with caution and may not be directly transferable to larger defects or patients with impaired healing.

## Author Contributions

S.P.B.: Conceptualisation, funding acquisition, investigation, methodology, project administration, visualisation, writing – original draft, writing – review and editing. A.C.F.: Data curation, methodology, project administration, investigation, visualisation, writing – original draft, resources, writing – review and editing. F.J.S.: Conceptualisation, methodology, visualisation, formal statistical analysis, writing – original draft, writing – review and editing. L.M.: Investigation, writing – review and editing. R.M.F.: Conceptualisation, resources, writing – review and editing. S.H.: Software, visualisation, validation, writing – review and editing. R.E.J.: Conceptualisation, funding acquisition, project administration, resources, supervision, writing – review and editing. D.S.T.: Conceptualisation, data curation, funding acquisition, methodology, project administration, supervision, writing – review and editing.

## Funding

The study received financial support through a grant from the Osteology Foundation (Grant No. 22‐036) and from the Clinic of Reconstructive Dentistry at the Center of Dental Medicine, University of Zurich. Additionally, material support was provided by Geistlich Pharma AG through a material transfer agreement covering the products used in the study. We gratefully acknowledge the support of Dentavis AG through Jeroen de Gier (Owner, Member of the Board of Directors; Partner Manager; Sales Manager for Central Switzerland) for providing the centrifuge used in PRGF preparation and the blood collection kits, which facilitated the implementation of the study protocol. We also thank Dr. Richard Miron, Lead Educator and Researcher at Advanced PRF Education and Adjunct Visiting Faculty in the Department of Periodontology at the University of Bern, for his valuable support in providing the centrifuge used for PRF preparation.

## Conflicts of Interest

Dr. Bienz reports personal fees from International Team for Implantology, personal fees and non‐financial support from Straumann Holding AG, grants and non‐financial support from Geistlich Pharma AG, non‐financial support from Thommen Medical AG and non‐financial support from Nobel Biocare, outside the submitted work. Dr. Thoma reports grants from Geistlich Pharma AG, as well as personal fees from International Team for Implantology and Straumann Holding AG. He receives non‐financial support from Straumann Holding AG, Geistlich Pharma AG and Thommen Medical AG and Nobel Biocare outside the submitted work. Dr. Jung reports grants and personal fees from International Team for Implantology, grants and personal fees from Straumann Holding AG, grants and personal fees from Vita, grants and personal fees from Henry Schein, grants from Osteology Foundation and personal fees and non‐financial support from Geistlich Pharma AG, outside the submitted work. He receives non‐financial support from Thommen Medical AG and Straumann Holding AG. He is a consultant for Geistlich Pharma AG, Henry Schein, Vita and TRI Dental Implants, and is an executive board member of the Osteology Foundation. Dr. Strauss, Dr. Fischer, Dr. Dr. Ferrari, Miss Hitz and Dr. Mancini report no conflicts of interest.

## Supporting information


**Figure S1:** Pain perception (NRS scale on the vertical axis) over time. The horizontal axis presents the number of days post surgery. CS, collagen substitute; PRF, platelet‐rich fibrin; PRGF, plasma rich in growth factor; control, spontaneous healing.
**Figure S2:** On the vertical axis, the probability of a patient in a specific condition (e.g., Visit—group) shows a higher NRS pain than other randomly selected from the total sample. CS, collagen substitute; PRF, platelet‐rich fibrin; PRGF, plasma rich in growth factor; control, spontaneous healing.
**Table S1:** Remaining open wound (%) by group: results of Wald's test corrected by Bonferroni. CS, collagen substitute; PRF, platelet‐rich fibrin; PRGF, plasma rich in growth factor; control, spontaneous healing; *p < 0.05.

**Supporting Information:** Appendix A.

## Data Availability

The data that support the findings of this study are available from the corresponding author upon reasonable request.
