# Supplementary material for: Wound Healing Patterns of PRF and PRGF Versus a Collagen‐Based Substitute and Spontaneous Healing—A Randomised Controlled Clinical Trial Assessing Wound Closure of an Experimental, Standardised Palatal Defect
Source: J Clin Periodontol. 2026 Aug 5;53(10):1565–74. doi: 10.1111/jcpe.70164 (PMC13581469; doi:10.1111/jcpe.70164)
Supplement: Supplementary file 1 [file JCPE-53-1565-s002.pdf]

Appendix A

Information on Exclusion Criteria

Any participant presenting with one or more of the following exclusion criteria was excluded from the study: history of surgical procedures at the palate within the past 3 months; uncontrolled diabetes; known coagulation disorders; use of medications affecting blood coagulation; smoking more than 10 cigarettes per day; self-declared pregnancy or breastfeeding at the time of inclusion or an intention to become pregnant during the study period; known allergies to local anaesthetics or collagen-based materials; discomfort with blood collection or the use of PRF or PRGF membranes; suspected or known non-compliance; substance or alcohol abuse; or any condition preventing adherence to study procedures, such as language barriers, psychological disorders or dementia. Furthermore, individuals were excluded if they had participated in another clinical trial involving an investigational drug within the 30 days preceding or during the course of the present study, if they had previously been enrolled in the current study or if they were the investigator, a family member of the investigator, an employee or otherwise in a dependent relationship to the study staff.

Randomisation

Prior to the intervention, an initial randomisation determined whether the first two blood collection tubes would be used for PRF or PRGF preparation. Allocation of the wound treatment groups followed a computer-generated randomisation list. Study monitors prepared sealed envelopes containing the assignment information, which were opened by the investigator during the surgical procedure, just before the creation of the four palatal defects.

Detailed PRGF Protocol

Two tubes of 9 mL each were collected, depending on the surgical procedure. The samples were centrifuged for 8 min at 580–600 RCF following the manufacturer's recommendations, resulting in separation into three layers. The supernatant plasma was divided into two fractions: Fraction 1 (F1), which is rich in fibrin and platelets, and Fraction 2 (F2), which contains a high concentration of growth factors and is located just above the buffy coat. The F1 and F2 fractions were separately extracted using a plasma transfer device and transferred into new tubes. To activate the plasma and neutralise the anticoagulant, 10% calcium chloride was added in a ratio of 2 units per 1 mL of plasma. Both fractions are incubated in the Plasmaterm H oven at 37°C. While F1 would form a fibrin barrier, it was not used in this study. F2, after activation, formed a fibrin clot within approximately 8–10 min.

Detailed PRF Protocol

For the PRF collection, glass Eppendorf tubes without silica coating were used (BD Vacutainer, red top) and immediately centrifuged in a Bio-PRF horizontal centrifuge at 400 RCF for 12 min. This process concentrates fibrinogen in the upper fraction of the tube, where it is converted by thrombin into a fibrin matrix, forming a platelet-rich clot located between the acellular plasma layer above and the red blood cell layer below. Immediately after centrifugation, the clot was removed and compressed into a membrane (Kobayashi et al. 2016).

Statistical Analysis

The percentage of open wound showed an approximately normal distribution for most groups and timepoints, except for the last two visits where values clustered around zero. Pain scores did not meet the normality assumption at any timepoint. The primary endpoint, percentage of open wound, was analysed using generalised estimating equations (GEEs) to account for within-subject correlation between defects. Treatment group, wound location and time were included as independent factors. Adjusted mean differences between each treatment group (PRF, PRGF and CS) and the control (spontaneous healing) were estimated with corresponding 95% confidence intervals and p-values. Pain scores (NRS) were analysed using nonparametric methods. The Brunner–Langer model for longitudinal data was applied to evaluate within-subject effects of treatment, time, location and their interactions, using ANOVA-type statistics (ATS). Because this rank-based approach does not provide conventional effect size measures such as Cohen's d, effect magnitude was described using differences in relative treatment effects (RTEs), whereby larger differences between the most extreme factor levels indicate stronger effects. When required, pairwise comparisons were performed using the Wilcoxon test.

Sample Size Calculation

The sample size was calculated using G*Power software based on the Wilcoxon signed-rank test, drawing on data from a previous publication (Bienz et al. 2024). To detect a large effect size ($d = 0.7$) with 80% power, assuming a standard deviation of 10 and a significance level of $\alpha = 0.05$, a total of 19 participants were required. To account for potential dropouts, the sample size was increased to 20 participants.
