# Supplementary material for: Wound Healing Patterns of PRF and PRGF Versus a Collagen‐Based Substitute and Spontaneous Healing—A Randomised Controlled Clinical Trial Assessing Wound Closure of an Experimental, Standardised Palatal Defect
Source: J Clin Periodontol. 2026 Aug 5;53(10):1565–74. doi: 10.1111/jcpe.70164 (PMC13581469; doi:10.1111/jcpe.70164)
Supplement: Supplementary file 2 [file JCPE-53-1565-s001.docx]

# Supplement

 ***Supplement Figure 1:*** *Pain perception (NRS scale on the vertical axis) over time. The horizontal axis presents the number of days post-surgery. CS = Collagen substitute; PRF = platelet rich fibrin; PRGF = plasma rich in growth factor; control = spontaneous healing.*

***Supplement Figure 2:*** *On the vertical axis, the probability of a patient in a specific condition (e.g. Visit – group) shows a higher NRS pain than other randomly selected from the total sample. CS = Collagen substitute; PRF = platelet rich fibrin; PRGF = plasma rich in growth factor; control = spontaneous healing.*

|  | **CS** | **PRF** | **PRGF** | **Control** |
| --- | --- | --- | --- | --- |
| **CS** |  | 1 | 0.126 | **0.042*** |
| **PRF** |  |  | 0.289 | **0.018*** |
| **PRGF** |  |  |  | 1 |
| **Control** |  |  |  |  |

***Supplement Table 1:*** *Remaining open wound (%) by group: results of Wald´s test corrected by Bonferroni. CS = Collagen substitute; PRF = platelet rich fibrin; PRGF = plasma rich in growth factor; control = spontaneous healing; * = p<0.05.*
